# Exploring drug coverage variability within districts: A CES approach to investigate treatment gaps in Mozambique’s schistosomiasis program

**DOI:** 10.1371/journal.pntd.0013751

**Published:** 2025-12-01

**Authors:** Jake D. Mathewson, Sergio R. Tomas, Andreas Nshala, Muhammed Semakula, Gertrudes Machatine, Marilia E. Massangaie, Henis M. Sitoe, Wilma A. Stolk, Oluwafemi J. Ifejube, Ente J.J. Rood

**Affiliations:** 1 Department of Epidemiology, KIT Royal Tropical Institute, Amsterdam, The Netherlands; 2 Crown Agents, ASCEND Mozambique Country Team, Maputo, Mozambique; 3 Crown Agents, ASCEND East Africa Regional Coordinator, Dar es Salaam, Tanzania; 4 Ministério da Saúde de Moçambique, Maputo, Mozambique; 5 Department of Public Health, Erasmus MC, University Medical Center Rotterdam, Rotterdam, The Netherlands; Seoul National University College of Medicine, KOREA, REPUBLIC OF

## Abstract

**Background:**

Coverage Evaluation Surveys (CES) are tools used by NTD control programs to assess drug coverage after mass drug administration (MDA), typically conducted at the district level. However, little research has explored how this district-level approach may mask uneven drug coverage within districts. Such uniform reporting may obscure gaps at smaller administrative levels, potentially limiting the effectiveness of MDA efforts. This study examines CES findings from a 2021 survey in Mozambique investigating coverage at the sub-district level to better understand heterogeneity in drug coverage, while also exploring other actionable survey data, like reasons for not taking drugs, to better understand why coverage gaps and discrepancies with reported coverage may have occurred.

**Methods:**

A CES was conducted in May of 2021 to assess district level drug coverage following schistosomiasis MDA in Nov-Dec 2020 in 10 districts in northern Mozambique. Following data collection, administrative post-level classifications were added retroactively by linking GPS data from the survey to spatial shapefiles, enhancing the analysis resolution. Programmatic coverage estimates were then calculated as the proportion of eligible individuals who reported taking praziquantel in each administrative unit. Finally, an ANOVA model was applied to examine the variance in drug coverage across province, district, and administrative post level, with cross tabulation exploring sub-district level variation.

**Results:**

Effective programmatic coverage (≥75%) was achieved with 95% confidence in five districts (Macossa, Majune, Mandiba, Maua, and Nipepe). Coverage discrepancies between surveyed and reported data were observed in nine out of ten districts, highlighting potential inconsistencies in routine reporting. ANOVA modeling showed that 49% of the observed variation in drug coverage could be explained by provincial, district, and administrative post-level differences. The ANOVA results, along with cross tabulation of coverage among administrative posts together highlight the likelihood of drug coverage variability within districts. Analysis of non-participation revealed that absence from the community at the time of MDA and lack of awareness of the campaign were the leading reasons for not taking the drug, particularly in urban districts.

**Conclusions/Significance:**

This study highlights how CES can be used to detect heterogeneity of coverage within districts while simultaneously identifying behavioral and operational barriers to participation. Integrating sub-district level spatial analysis with contextual data on reasons for non-uptake can support in the identification of local treatment gaps, which may be integral to tailor approaches to improve MDA participation in future campaigns as national schistosomiasis programs are increasingly urged to conduct MDA at a sub-district level. These findings suggest that more granular data collection within CES could better inform program adjustments and resource allocation.

## Authors summary

Coverage Evaluation Surveys (CES) are widely used by neglected tropical disease (NTD) control programs to assess drug coverage following mass drug administration (MDA) campaigns. These surveys have historically been conducted at the district level, under the assumption that district-wide results reflect overall coverage across target populations. However, little research has examined the degree of uniformity in drug coverage within districts, potentially overlooking sub-district-level coverage gaps that could impact disease control efforts. This lack of granular analysis can conceal disparities in MDA reach and uptake across smaller administrative units, such as the administrative posts examined in this study, which could provide more actionable insights for program improvement. By examining CES findings at finer geographic levels, this study aims to shed light on within-district variability in coverage, ultimately enhancing the precision and responsiveness of MDA campaigns for schistosomiasis control in Mozambique. By identifying and addressing the specific reasons for not taking medication, this study further underscores the importance of understanding barriers to drug uptake at local levels. This level of contextual insight provides actionable information that can inform tailored interventions to target these obstacles effectively. Moreover, the study’s approach to assessing drug coverage within smaller administrative units has implications beyond Mozambique, offering a potential model for other schistosomiasis-endemic regions. Adopting a similar framework in varied contexts could reveal hidden coverage gaps within larger administrative units, supporting more precise targeting of resources and ultimately strengthening the impact of MDA programs globally.

## Introduction

Schistosomiasis is a devastating and preventable disease caused by parasitic blood flukes of the genus *Schistosoma* [1]. The World Health Organization estimates that over 250 million people globally are at risk of getting the disease in 51 moderate to high transmission countries, predominantly in sub-Saharan Africa [1]. Schistosomiasis can cause a range of severe morbidities and secondary diseases depending on the species of schistosome, the two predominant species in sub-Saharan Africa being *Schistosoma haematobium* and *Schistosoma mansoni* [2]. While both *S. haematobium* and *S. mansoni* are endemic in Mozambique, *S. haematobium* is considerably more common [3]. Infection with S. haematobium can cause urogenital disease and dysfunction including dysuria, haematuria, reproductive problems, renal failure, and cervical and bladder cancers [4]. Infection with S. mansoni is associated with intestinal and hepatic and splenic disease, including periportal fibrosis, portal hypertension, and upper gastrointestinal bleeding [5,6]. In Mozambique, schistosomiasis is a major public health problem, with prevalence rates historically exceeding 50% in several northern provinces [7–9]. As of 2022, more than half of the country’s population lived in districts requiring treatment for schistosomiasis [10]. Schistosomiasis is classified as a Neglected Tropical Disease (NTD) due to its disproportionate impact on impoverished and marginalized populations, and persistent funding constraints and lack of research prioritization which continue to hinder progress toward its elimination [11,12].

The primary control strategy to reduce schistosomiasis transmission and morbidity associated with disease progression is mass drug administration (MDA) with the preventive chemotherapy (PC) drug praziquantel [13,14]. MDA entails the large-scale distribution of PC drugs to at risk populations across pre-determined geographic areas regardless of infection status [15]. The goal of MDA is to reach and treat a sufficiently large proportion of the population at risk that it will drive down disease prevalence and ultimately reduce transmission [12]. MDA is a commonly used public health intervention, a cornerstone control strategy for several NTDs (collectively termed PC-NTDs) [16], and a supplementary intervention for better researched diseases like malaria [17]. The success of PC-NTD control campaigns hinges on a program’s capacity to consistently and effectively distribute PC drugs through MDA [18,19]. Disease modeling and quantitative analysis of past MDA campaigns have demonstrated that for schistosomiasis, maintaining drug coverage above 75% of the target population is necessary to interrupt disease transmission and ultimately limit the need to continue mass treatment [20,21]. Target populations for schistosomiasis MDA have historically included school aged children (SAC) between 5 and 14 years old and adults at high risk of infection. However, based on updated WHO recommendations, target groups have now expanded to include children above 2 years old and women of reproductive age [5,14,21].

While the importance of consistently exceeding the target of 75% drug coverage in MDA campaigns for schistosomiasis control is widely understood, NTD control programs must contend with numerous challenges in the distribution of PC that can inhibit programs from meeting treatment targets. These can include operational challenges like accessing remote populations, community willingness to take drugs, limited resources for NTD control programs to carry out the interventions [22–24], and consistently in low resource settings like Mozambique, challenges related to the reporting of drug coverage [25–29]. Challenges to reporting include poorly documented shifts in population and reliance on outdated census data [30] and political pressures to meet drug distribution targets, which have contributed to past incidents of deliberately inflated drug coverage figures [30–34]. Common use of conventional paper-based data collection methods, instead of using digital tools, may further inhibit efforts to accurately and efficiently report these coverage figures, as paper-based data take more time to be re-entered digitally. Re-entered data can be failure prone, both through errors at the time of entry and because it is often aggregated to larger geographic extents, losing the granularity that can support more targeted sub-district coverage review and MDA planning [35–38].

With limitations in access to reliably reported drug coverage figures following MDA, household drug coverage evaluation surveys (CES) have been demonstrated to be a vital tool that can provide programs with more accurate coverage figures than what is reported [30,32,39–41]. Use of routine and integrated CES to improve MDA delivery has even been attributed to past successes in NTD elimination efforts in some settings [41]. CES are used to 1) determine if effective coverage has been met, to 2) validate reported coverage figures, 3) detect problems with distribution systems, 4) understand community attitudes toward MDA and drug consumption and 5) measure coverage in specific populations [42]. Additional data can furthermore be collected through coverage surveys that can provide information surrounding determinants of health. Without accurate drug coverage estimation, programs risk delivering ineffective MDA, potentially necessitating additional, costly rounds of treatment [13]. This strains the already limited resources of chronically underfunded programs, can lead to participation fatigue among community members, and may heighten the risk of drug resistance for certain PC NTDs [43]. While WHO guidelines recommend the frequent use of independent surveys and context specific survey tools to verify and validate reported MDA coverage results, they do not explicitly state that CES should be conducted following every round of MDA [44]. For CES, the WHO provides recommendations and protocols to guide participating programs in acquiring the most accurate assessment of drug coverage possible [42,44].

While CES are vital monitoring tools to ensure that MDA campaigns for schistosomiasis control are reaching their 75% target coverage threshold, there are limitations in how they are presently used [45]. Firstly, their aggregation of drug coverage to the level of a district their aggregation of drug coverage data to the level of a district “precludes estimates in small but programmatically important sub-districts” [45]. Although CES provides insight into coverage at the district level, little is known about its accuracy and utility for evaluating heterogeneity within sub-district areas, which could exclude areas with lower-than-expected coverage or high-risk populations. There is considerable uncertainty regarding the reliability of reported coverage data when compared to CES estimates. Discrepancies between CES findings and reported figures have been documented in numerous studies [45], yet surveys often fall short in identifying the factors that contribute to these differences. Finally, there is a paucity of research exploring the use of CES at sub-district level, detailing challenges and limitations to support future efforts in monitoring sub-district areas. This is especially relevant since the WHO now advocates for implementing PC at the sub-district level to account for the heterogeneity of disease prevalence [35,46]. In Mozambique, the units of official units administration from largest to smallest are province, district and administrative post [47]. At the time of this survey, Mozambique was implementing MDA distribution at the district level, with coverage assessments conducted at the same geographic scale. With substantial transmission of schistosomiasis still occurring in Mozambique [9,48] and other schistosomiasis endemic settings across sub-Saharan Africa [12], research is indicated to determine if CES can be used to better identify areas within districts not meeting effective coverage thresholds, and subsequently facilitate the targeted distribution of PC drugs.

This study aims to address several key knowledge gaps in CES implementation to better inform programs of coverage gaps, ultimately improving subsequent MDA interventions. Focusing on a household CES conducted across three provinces in Mozambique with historically high schistosomiasis prevalence, the study investigates: 1) factors contributing to discrepancies between reported and CES-derived programmatic coverage estimates, 2) the value of examining rationale for not taking drugs to identify potential barriers to drug uptake, and 3) the presence and extent of heterogeneous drug coverage within districts, which may help inform sub-district-level planning now mandated under WHO guidelines.

## Methods

### Ethical considerations

At the onset of each survey, verbal consent was obtained from the head of household and individual survey participants prior to questionnaire administration. For children, verbal consent was first obtained from a parent or guardian, followed by verbal assent from the child, who also had the option to decline participation. Caregivers were permitted to respond on behalf of children under 10 years of age. At all points in the data collection and management process, participant anonymity was maintained, and data was stored and shared in compliance with GDPR standards. Rented phones used password protected Kobo applications, which sent and cleared all survey forms once internet connectivity was accessible.

The CES was conducted under the activities of the ASCEND program, and the subsequent analysis considered as part of ongoing programmatic activities for the prevention, control, and elimination of schistosomiasis. On 14 April, 2023, the National Directorate of Public Health of Mozambique issued a waiver, note no. 920/OOZ/DNSP/2023, to agree that dissemination of the results for the survey that took place in 2021 did not need further ethical approval.

### Study design

A cross sectional community based household survey was conducted in Mozambique in May, 2021 examining schistosomiasis and lymphatic filariasis (LF) drug coverage following MDA in November 2020 in accordance with WHO guidelines [42].

### Survey area

**Fig 1:**
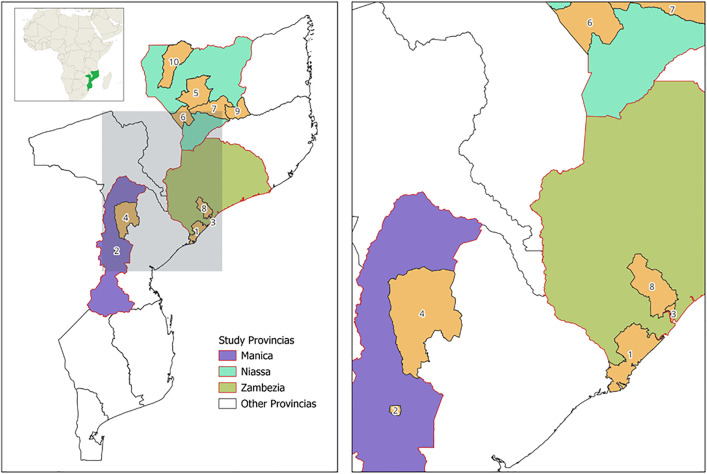
Study area of the ten districts across three provinces (Manica, Niassa, and Zambezia) in Mozambique where the coverage evaluation survey was implemented in May 2021, following mass drug administration for schistosomiasis. The map on the right provides a zoomed-in view to better visualize smaller urban districts. Districts are labeled numerically in alphabetical order: 1) Chinde, 2) Cidade Chimoio, 3) Cidade Quelimane, 4) Macossa, 5) Majune, 6) Mandimba, 7) Maua, 8) Nicoadala, 9) Nipepe, and 10) Sanga. The maps were created in QGIS 3.30.1 using open-source spatial data from GADM.org (https://gadm.org/license.html).

The CES was conducted in 15 of the 31 districts of Mozambique where MDA took place in November 2020. The CES was designed to examine schistosomiasis, LF, and joint schistosomiasis/LF MDA campaigns. This study, however, more specifically examines schistosomiasis MDA outcomes. The study investigated coverage for schistosomiasis MDA among school aged children (SAC) between 5 and 14 years old in the 10 districts across 3 provinces, shown in Fig 1. Due to COVID-19 control measures in place during this time, the majority of MDA campaigns were home-based.

### Sample design

The primary sampling unit for the CES was an administrative district. Of the 31 districts that implemented MDA during the November 2020 MDA campaign, a sample of 15 was selected to be included in this CES due budgetary constraints. Districts were selected by first stratifying the type of MDA campaign: LF MDA, schistosomiasis MDA, or combined LF and schistosomiasis MDA. Within each stratum, five districts were selected randomly using a random number generator. An exception was made for the LF only stratum, where all four eligible districts were purposefully included in the survey, in addition to one more random one from the combined group. This study focuses specifically on the 10 districts where coverage for schistosomiasis was assessed. The sample design for the survey within each district was conducted in accordance with the WHO Preventive Chemotherapy field manual for implementation [44].

To determine the minimum sample size for individuals in each district, the following formula was applied in accordance with the WHO field manual and COR NTD survey builder tool:

$\text{n }=\frac{\left(\text{DEF}({\text{Z}}_{\propto /\text{2}}^{\text{2}}(\text{p})(\text{1}-\text{p})\right)}{\delta^{\text{2}}(\text{1}-\text{r})}$

For this survey, a design effect (DEF) of 4 was considered since there was limited information about previous surveys and assumed heterogeneity in coverage within clusters. This is also recommended as the default DEF in the WHO field manual [44]. Expected coverage rate (p) was calculated by taking off 15% of reported coverage rate of each district above a 50% epidemiological coverage rate. For districts below 50%, a 50% default rate was used. A suggested default of ±5% was used for the desired precision (δ). An alpha value (α) of.05 was used corresponding to 95% confidence intervals was used. Finally, this survey used a 10% non-response rate (r) corresponding to the survey population from whom data would not be obtained due to absenteeism.

The number of households necessary to survey to reach the minimum sample size was calculated using population projections, obtained from the Mozambique National Institute of Statistics from the latest census estimation (2017) [47]. Since the survey was targeting a subset of the population, people aged 5 – 14 years old (SAC), the national proportion of the population in this age demographic (27%) was multiplied by the average household size in Mozambique (4.5 persons) to determine the number of SAC expected in each household (1.2). The sampling size distribution can be visualized in S1 Table. For each district, 30 villages, or enumeration areas (EAs) were randomly selected with support from coverage evaluation survey sample builder v2.11 [49]. The required sample size per EA was defined using probability proportional to estimated size (PPES), resulting in a self-weighing representative samples in each district. A sample frame was constructed based on 3 levels of administration: district, administrative posts, and localities.

After listing the households within the EA, the survey team created 2 segmented community maps, each containing 50 households, to facilitate field logistics. Local guides assisted in refining these maps, ensuring that the paths taken by data collectors covered the required number of households. To introduce further randomization, a coin toss was used to determine which segmented map the data collectors followed. One segment was randomly selected in each EA. Within a segment, the HHs were randomly sampled (every n^th^ household) using a systematic random sampling approach. If nobody was in a household at the time of the survey and not expected to return later that day, data collectors would proceed to the next selected house. A replacement household was not needed as the sample size was inflated to account for ineligibility.

### Data collection and management

CES responses were collected in May, 2021, 6 months after the previous MDA, over a period of 15 days. Field teams collected and entered data digitally using Kobo Toolbox on rented Android phones feeding into a central data repository [50]. To ensure data quality and standardization of methods across provinces, all field team members completed a 3-day training where they completed mock surveys and piloted the data collection tools. This included training on collecting GPS coordinates outside of houses, which were only to be saved if the head of household consented to participating in the survey. Access to survey software and central data repositories were limited to three experienced data managers to ensure data security.

The questionnaire included individual questions for SAC about whether the drug was taken, reason for not taking the drug, adverse events, and other topics listed in S2 Table. Heads of household were asked a number of questions including highest level of education, occupation, distance to nearest health care facility, questions related to disease specific knowledge attitude and practice as well as water sanitation and hygiene. Data collected from heads of household were linked to each school aged child living there eligible to respond to the survey. Participants were included if they were aged between 5 and 14 and residing in the selected village at the time of the MDA. Participants were excluded from the survey if they refused participation, were not a resident of one of the selected villages or were pregnant at the time of the MDA.

All surveys and tools were subject to testing and quality control during enumerator training sessions and piloted prior to survey implementation. MDA reported coverage data was supplied by the MoH of Mozambique and is additionally available in the DHIS2 ASCEND database.

### Data analysis

Drug coverage for the CES was estimated by dividing the number of individuals who reported swallowing praziquantel by the total eligible participants surveyed within each Implementation Unit (IU). These estimates, along with associated confidence intervals, demographic cross-tabulations and rationale for not taking the drug were analyzed using survey commands in Stata 15.1 [51].

Spatial analysis was conducted using QGIS software [52] to map drug coverage estimates and explore geographical patterns in coverage. Administrative post data were assigned to individuals where GPS coordinates were collected based on shapefile data obtained from GADM.org [53], allowing participants to be categorized by sub-district level geography. R version 4.1 [54–56] was later used for creating the visual plot for drug coverage by administrative post, which as for district level coverage, was analyzed in STATA. Administrative posts were chosen over enumeration areas for analysis due to their alignment with programmatic boundaries that may serve as future implementation units for MDA distribution. After province and district, administrative posts are the smallest unit of administration in Mozambique formally recognized by government programs [57]. Within administrative posts are less formally organized localities, and within them, communities.

To assess the variability in drug coverage across different levels of administrative geography, an Analysis of Variance (ANOVA) model was applied to evaluate the proportion of variance in drug coverage attributed to provinces, districts, and administrative posts. The ANOVA model was implemented to quantify how much of the observed variance in drug coverage could be explained by the different administrative levels which they were analyzed: provinces, districts, and administrative posts.

## Results

### Demographics

We surveyed 7,280 school aged children in 2,370 households who met the inclusion criteria and consented to participate in the CES. In Table 1, demographic data are presented, including the number of surveyed participants in each district with respective age and gender distribution. Of the total group surveyed, data collectors were able to gather GPS coordinates for 3,788 participants, representing 52% of the total participants. Data collectors in Manica province, particularly in Cidade Chimoio, reported functionality issues collecting coordinates that were attributed to the condition of the phones being used to enter data. This led to gaps in collected coordinates across the province, and no coordinates collected in Cidade Chimoio District, as shown in Table 1.

**Table 1:** Demographics of surveyed participants.

Province	District	School aged children surveyed (n)	Total Households (n)	age (mean, CI)	males (%)	Coordinates collected (n)
Manica	Cidade Chimoio*	899	264	9.0 (8.8 - 9.3)	49	0
Manica	Macossa	362	137	10.2 (9.9 - 10.5)	41	148
Niassa	Majune	847	330	9.4 (9.2 - 9.6)	49	205
Niassa	Mandiba	591	228	7.4 (7.3 - 7.6)	57	233
Niassa	Maua	874	189	8.9 (8.7 - 9.1)	55	795
Niassa	Nipepe	1116	340	8.5 (8.3 - 8.6)	58	536
Niassa	Sanga	1667	534	9.3 (9.1 - 9.4)	56	964
Zambezia	Chinde	254	127	10.6 (10.2 - 11.0)	53	241
Zambezia	Cidade Quelimane*	311	103	10.4 (10.0 - 10.9)	45	309
Zambezia	Nicoadala	359	121	10.6 (10.2 - 10.9)	53	357
	**Total**	**7280**	**2373**	**9.1 (9.1 - 9.2)**	**53**	**3788**

This table shows the number of eligible school aged children surveyed in each district. Included is the average age of survey participant, distribution of males and female, and as well as the number of coordinates that survey teams were able to collect in each respective district. *Represents the urban districts.

**Fig 2:**
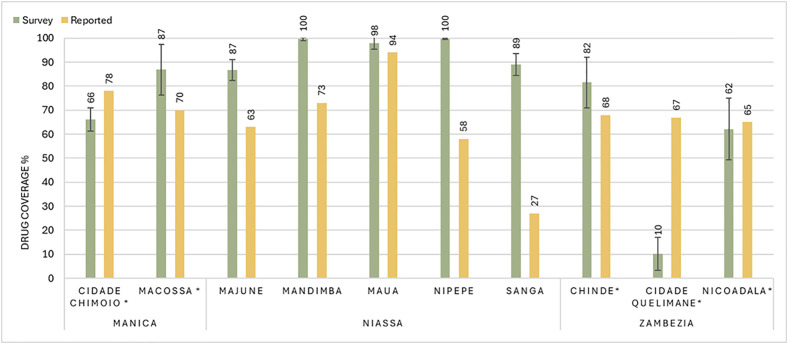
Reported vs Surveyed Coverage: Mean survey estimates of drug coverages by district (left bar) compared with the reported drug coverage (right bar) of that district. The Error bars represent 95% confidence intervals surrounding coverage estimates. Districts are alphabetized in each of their corresponding provinces, which are written below the district names. - - - - represents the 75% target coverage threshold for effective schistosomiasis MDA * Indicates districts that also had LF MDA campaigns.

Fig 2 shows the survey estimates of programmatic drug coverage in each study district, and indicates whether each district exceeded the 75% effective target coverage threshold. Based on the survey results, effective programmatic coverage (≥75%) was achieved with 95% confidence in five districts: Macossa, Majune, Mandiba, Maua, and Nipepe. Statistical significance was assessed by comparing reported coverage to the surveyed 95% confidence intervals. Reported coverage differed significantly from surveyed coverage in all districts except for Nicoadala. Some districts were found to have a much lower surveyed coverage than what had been reported, as seen in the two urban districts: Cidade Quelimane and Cidade Chimoio. Alternatively, survey estimates of drug coverage were found to be higher than what had been reported in districts across Niassa and Manica provinces, with some variation. No statistical differences were found between coverage in males and females in any of the study districts.

Table 2 shows reasons for not taking praziquantel among the participants who responded ‘no’ to the survey question: did you swallow the drug at the time of the MDA. Participants were able to provide as many of the multiple-choice answers as they wanted and could additionally list ‘other’ reasons outside of the main selection options. Among participants who selected ‘other,’ the key themes for not participating included a lack of sufficient information, a belief that they were too old to be eligible for treatment, or uncertainty about the reason for non-participation. The main reasons that the surveyed school aged children indicated that they did not take the drugs during MDA was that 1) drug distributors did not come to their locations, 2) that they were not in the community at the time of the MDA, or 3) that they were unaware of the MDA. Not being in the community at the time of the MDA was noted to be particularly high (74%) in Cidade Quelimane, an urban district with the low surveyed coverage.

**Table 2:** Reasons for not taking drugs.

District	Respondents (n)	Too far away (%)	Unaware of MDA (%)	Underage (%)	Did not come to location (%)	Not in the community at the time of MDA (%)
Chinde	49	31	49	4	12	35
Cid Chimolo*	307	7	1	0	60	28
Cid Quelimane*	272	5	4	0	6	74
Macossa	49	6	35	45	2	4
Majune	113	11	1	4	43	33
Mandiba	1	0	0	0	100	0
Maua	14	21	29	21	21	21
Nicoadala	135	4	66	0	56	10
Nipepe	4	0	0	25	100	0
Sanga	185	63	6	1	10	19
**Total**	**1129**	**15**	**19**	**10**	**41**	**23**

Survey respondents who answered that they did not take the drug were able to select from reasons why they did not take drugs, and/or provide additional reasons under the ‘other’ category. Respondents were able to provide more than one reason.

* Indicates urban districts.

Among participants who reported swallowing the drug, 87.9% of all participants reported taking the drug at home (Table 3). This was predominantly the case in several districts in Niassa province, while other districts showed greater variation in drug coverage delivery, including a mix of home-based, school-based, healthcare facility-based, and community central point MDA.

**Table 3:** Venue location for drug administration, as per participants who reported swallowing the drug.

Province	District	Total swallowed	Central point in the community (%)	Health facility (%)	Home (%)	School (%)
Zambezia	Chinde	205	34.6	54.6	7.3	2.4
Zambezia	Cidade Quelimane	32	12.5	3.1	65.6	3.1
Manica	Cidade Chimoio	595	0.5	4.4	78.3	16.8
Manica	Macossa	319	0.3	0.6	68.7	30.4
Niassa	Majune	735	4.9	1.6	92.7	0.8
Niassa	Mandiba	595	0.0	0.2	99.2	0.0
Niassa	Maua	859	0.7	0.3	95.2	3.6
Zambezia	Nicoadala	223	28.3	18.8	17.5	35.4
Niassa	Nipepe	1117	0.7	0.3	98.9	0.0
Niassa	Sanga	1484	0.7	0.6	98.7	0.0
	**Total**	**6164**	**3.3**	**3.4**	**87.9**	**5.2**

### Geographic variation in drug coverage

The collection of GPS coordinates during the survey allowed for the analysis of drug coverage and other results at smaller levels of administration within districts, in Mozambique known as administrative posts (posto administrativo). As shown in Table 1, GPS coordinates were collected for 3,788 of the 7,280 participants. An analysis of variance (ANOVA) model was conducted to investigate the proportion of variance in drug coverage explained by geographic levels: province, district, and administrative post. The model suggests that approximately 49.2% of the variance in drug uptake (R² = 0.49, Adj R² = 0.48) can be explained by geographic variation across province, district, and administrative post levels. All geographic levels, province, district, and administrative post, were statistically significant (p < 0.001), indicating they contribute to variation in drug coverage. Within the model, province had the largest effect on variance (F = 54.28), followed by administrative post (F = 11.56) and finally district (F = 6.51).

**Fig 3:**
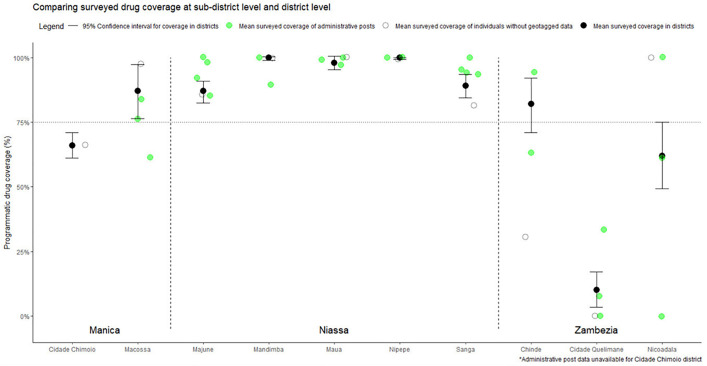
Comparing surveyed drug coverage of administrative posts with their underlying district averages. Many PA averages statistically differed from the mean drug coverage in underlying districts, demonstrating variation of drug covreage within districts. The average drug coverage amongst individuals who were not geotagged at the time of the survey, and therefor unable to be assigned any sub-district specification are displayed by open circles.

Fig 3 shows survey estimates of all administrative posts and how they compare with their respective district averages. A wide range in coverage is evident among the administrative posts within some of the districts, with many administrative posts exceeding or falling short of district level estimates.

## Discussion

This study assessed coverage of the drug praziquantel for the control of schistosomiasis across three provinces, ten districts, and twenty-six administrative posts in Mozambique. A survey of 7,280 SAC from 2,370 households evaluated the effectiveness of MDA campaigns. The CES data indicated that mean drug coverage exceeded the World Health Organization’s (WHO) effective target coverage threshold of 75% in seven out of ten districts surveyed. However, discrepancies emerged between estimated and reported coverage in nine of the ten districts, suggesting potential gaps in reporting accuracy. Additionally, an ANOVA model and cross-tabulation of administrative posts highlighted the likelihood of heterogeneity of drug coverage within districts.

The discrepancy between survey estimates and reported coverage in this study warrants further investigation. While discordance between CES estimates and reported coverage is not uncommon to find [45], it can be a useful step for refining MDA monitoring and guiding improvements in future campaigns. In Niassa’s Sanga district, CES coverage estimates were notably higher than reported figures, which may suggest potential issues with population denominators in reporting. Outdated or inaccurate population registers can contribute to both underreporting and overreporting of coverage figures. If programs do suspect that outdated population registers are affecting reporting, as may be the case in many districts in Niassa province, they can conduct a pre-MDA registration which has proven effective in other settings [58]. Out of date population registers can further challenge delivery of other essential health programs in Mozambique like vaccine distribution and malaria control, so it is important that programs flag areas where figures are likely to be incorrect.

CES may be programmatically useful not only to demonstrate *if* coverage was sufficient, but also to explore reasons *why* low coverage may have occurred. In our study, the stark contrast in reported coverage (70%) and surveyed coverage (10%) in Cidade Quelimane may suggest a gap in the process of distributing drugs, enumeration of drug recipients or underlying populations, or both. Urban districts like Cidade Quelimane often face unique challenges in drug distribution, including issues of population enumeration and logistical barriers [24], which may lead to over-reporting. Among participants in Cidade Quelimane district who did not take the drug, 74% cited being “not in the community at the time of the MDA” as their reason for non-participation. This finding could point to a common issue in urban areas, where transient and mobile populations may lead to discrepancies between MDA timing and the actual population present during the survey six months later [24]. In two nearby districts also within Zambezia province, Chinde and Nicoadala, 49% and 66% of non-participants, respectively, stated they were unaware that drug distribution was taking place. Such responses may be an indication to programs that enhanced social mobilization may be necessary to improve awareness ahead of future MDAs in these areas, although it should be considered that these responses come from a small sample size of individuals who had not taken the drug. Identifying these specific barriers through CES offers actionable insights for programs, emphasizing the need for targeted communication strategies that could increase drug uptake.

The collection of digital and GPS data and in the CES also allows for further investigation into populations to see if specific groups are being missed in drug distribution efforts. In the district with the lowest coverage, Cidade Quelimane, although more girls received the drug than boys, no significant differences in drug coverage by gender were found (nor in any other district in the survey). However, stratifying district wide coverage by administrative post, as seen in Fig 3, we can see variance in coverage estimates, potentially indicating to programs that coverage gaps are more marked in one part of the district than in others. As it can be hard to draw conclusions based upon aggregated district wide results, such stratifications among population groups and geographies may help programs investigate groups that are systematically missed by drug distribution efforts during MDA. Past research has presented evidence that certain groups have been shown to consistently abstain from drug treatments during MDAs, which poses a significant challenge to meeting the goal of eliminating the disease as a public health problem by 2030 [59]. Future surveys may want to include an additional question on whether participants have *ever* received drugs, to better identify these groups. However, such questions may be more prone to recall bias as they require respondents to remember events several years into the past.

An important consideration of this CES study is the exploration of variation in drug distribution within districts, shown in sub-district level analysis. Although this CES was not powered to make statistical inferences on the drug coverage of administrative posts, both analysis of the ANOVA model (Table 2) as well as the sub-district level stratification of surveyed coverage (Fig 3) highlight the likelihood of variance in drug coverage among administrative posts within the same districts. With the WHO now recommending targeted schistosomiasis MDA at the sub-district level, the need to determine feasible sub-district monitoring strategies is pressing [12].

Powering CES at sub-district level, while still surveying the same number of districts, would likely be significantly more resource intensive than conventional district level surveys. With finite resources available to most programs in NTD control, this may create a tradeoff: is it best to aim for very targeted coverage assessments where the sub-district level units of administration are adequately powered, or rather implement a survey with a larger geographic spread of districts?

This study presents one way where administrative post data may be programmatically considered, even when sufficient powering has not occurred to make strong statistical inferences. It may further demonstrate how even past surveys where geospatial data has been collected that can tie it to sub-district level administrative units, can be examined to prompt further hypotheses about how to improve coverage and tailor response in underperforming areas.

### Limitations

A primary limitation of this study is that it was not initially powered for analysis at the sub-district level, as the main objective was to assess drug coverage at the district level. Consequently, results at the administrative post level may not have the statistical rigor of a study explicitly designed with this unit of analysis in mind. Future studies could consider Bayesian approaches, which may offer advantages when working with smaller sample sizes or hierarchical data structures. Additionally, administrative post data and GPS coordinates were only available for a subset of the surveyed sample, limiting the consistency and granularity of spatial analysis across all surveyed areas. In some districts, the proposed sample size of participants was not reached due to time constraints and mobility challenges faced by field teams under COVID-19 control measures. However, the original sample size calculations incorporated a high design effect to account for potential fieldwork limitations, including lower than expected survey participation.

The use of different data collectors across districts may have influenced how reasons for non-participation in the MDA were interpreted and recorded. In future surveys, we recommend limiting respondents to selecting a single primary reason for non-participation to facilitate more standardized analysis. The timing of the survey, conducted six months after the MDA, may also introduce recall bias. However, this falls within the WHO’s recommended window of six months or less post-MDA [44] and previous studies on recall bias in CES have furthermore shown that recall accuracy can be maintained for up to one year post-MDA [39]. Despite this, future studies could benefit from a shorter interval to further minimize any potential recall bias. Findings from the five districts with integrated schistosomaisis/LF MDA are at increased risk of recall bias because of the two separate treatments distributed. Consent was limited to verbal consent from heads of households, verbal consent from parents and guardians and verbal assent from SAC. This approach was considered appropriate by Mozambique national teams in coordination with ethical committees, given the low-risk nature of the survey.

## Conclusion

The results from this study highlight how CES can be utilized not only to assess overall drug coverage at the district level but also to provide insights into factors contributing to sub-optimal coverage and to identify specific areas within districts where coverage may be lacking. Given the observed heterogeneity of drug coverage within districts, we recommend further exploration into methods of conducting CES at the sub-district level. Planning for this level of granularity should begin at the sampling stage to ensure adequate sample size within each administrative unit. Using digital surveys that capture GPS coordinates can further enhance data accuracy and facilitate sub-district level analysis of existing administrative areas or programmatically designed ecological zones. When collecting GPS data is not feasible, using structured dropdown menus for sub-district selections is advisable. Employing both methods would help verify locations and minimize ambiguities regarding boundary names or administrative areas.

This study adds to a growing body of evidence demonstrating limitations in reported drug coverage data. Routine implementation of CES alongside each round of MDA can provide valuable, programmatically actionable information, as presented in this study. CES is a relatively low-cost intervention, especially when compared to the high expense of MDA campaigns that require repetition [60]. We also recommend more explicit language in WHO guidelines explicitly advocate for conducting CES with *every* round of MDA. Adding this could encourage national programs to incorporate CES into routine monitoring, as many countries tend to align their control strategies with WHO recommendations [61].

Acknowledging drug coverage heterogeneity within districts is a critical first step toward strengthening schistosomiasis control strategies. By revealing both the spatial distribution of coverage gaps and the reasons for non-participation, CES offers programs a powerful tool to facilitate more tailored MDA programs. These findings underscore the value of incorporating CES into routine program evaluation to support more targeted and responsive schistosomiasis control efforts.

## Supporting information

S1 Table Coverage evaluation survey sample overview. (DOCX)

S2 Table Main survey themes. (DOCX)
