# Supplementary material for: Exploring drug coverage variability within districts: A CES approach to investigate treatment gaps in Mozambique’s schistosomiasis program
Source: PLoS Negl Trop Dis. 2025 Dec 1;19(12):e0013751. doi: 10.1371/journal.pntd.0013751 (PMC12677778; doi:10.1371/journal.pntd.0013751)
Supplement: S1 Table — (DOCX) [file pntd.0013751.s001.docx]

S1 Table .Coverage evaluation survey sample overview

| **Province** | **District (IUs)** | **Sample size per district** | **Cluster** | **HHs to be Interviewed per district** | **Expected Coverage used for n** | **Targeted Disease** |
| --- | --- | --- | --- | --- | --- | --- |
| Niassa | Mandimba | 773 | 30 | 172 | 85% | SCH |
| Niassa | Maúa | 871 | 30 | 194 | 85% | SCH |
| Niassa | Nipepe | 1377 | 30 | 306 | 72% | SCH |
| Niassa | Sanga | 1708 | 30 | 380 | 50% | SCH |
| Niassa | Majune | 1347 | 30 | 299 | 73% | SCH |
| Zambezia | Cid Quelimane | 871 | 30 | 194 | 85% | Both |
| Zambezia | Chinde | 871 | 30 | 194 | 85% | Both |
| Zambezia | Nicoadala | 964 | 30 | 214 | 83% | Both |
| Manica | Cid. Chimoio | 871 | 30 | 194 | 85% | Both |
| Manica | Macossa | 954 | 30 | 212 | 83% | Both |
