# Supplementary material for: Exploring drug coverage variability within districts: A CES approach to investigate treatment gaps in Mozambique’s schistosomiasis program
Source: PLoS Negl Trop Dis. 2025 Dec 1;19(12):e0013751. doi: 10.1371/journal.pntd.0013751 (PMC12677778; doi:10.1371/journal.pntd.0013751)
Supplement: S2 Table — (DOCX) [file pntd.0013751.s002.docx]

S2 Table . Main Survey Themes
